# The Ice-Bag in the Treatment of Pneumonia

**Published:** 1900-06-23

**Authors:** 


### THE ICE-BAG IN THE TREATMENT OF PNEUMONIA.

In a clinical lecture on pneumonia, recently delivered at the Liverpool Royal Infirmary, Dr. James Barr spoke highly of the advantages attending the use of the abdominal ice-bag as a means of lowering the temperature, and gave a special warning against the use of coal tar antipyretics for that purpose. He said he had tried the application of the ice-bag to the affected side as recommended by Dr. Lees, but while they afforded great relief to the pleuritic pain they never seemed to control the internal temperature, and he thought that they occasionally lowered the vitality of the part and thus tended towards the development of pneumonia. Ice cradles he regarded as a most unscientific proceeding, for air, being a very bad conductor of heat, its cooling effect was chiefly due to the evaporation produced. Thus a current of moderately warm, dry air would abstract much more heat than a cold atmosphere saturated with moisture, and would certainly be much more agreeable to the patient. The abdomen, however, afforded a large cooling surface, and he thought that the application of one or two ice-bags, sufficient to cover the whole abdomen, would lower the general temperature, and from the action of the cold on the splanchnic area would raise the general arterial pressure, and finally would diminish the frequency and increase the depth of the respirations. Before using this method in pneumonia he had observed its efficacy in a large number of cases of typhoid fever, and he had also observed that except in the groins, which can easily be protected with a little cotton wool, the abdomen is not very sensitive to cold, so that the method was not particularly uncomfortable to the patient. He then related a series of cases so treated with apparently the best results. He insisted, however, on this, that it was in the early stages of the disease that ice was useful. There is no use, he said, in attempting to arrest an inflammation which has reached its height. If the lung be in a stage of grey hepatisation, one should encourage resolution by warm poultices. Nor should ice be used in feeble old people, or in those with degenerate hearts, which cannot cope with much peripheral resistance. In very corpulent people, also, ice seemed to fail in removing excess of heat satisfactorily. We should have liked to have heard Dr. Barr's opinion as to the use of Leiter's tube in such cases, this apparatus often being more immediately available than ice, especially in country practice.

